# ANP32A interacts with LDHA to modulate glycolysis and ferroptosis in hepatocellular carcinoma

**DOI:** 10.3389/fonc.2026.1908459

**Published:** 2026-08-28

**Authors:** Bixia Chi, Zhi Cui, Xinyue Hou, Hongfang Ding, Linjun Li, Rui Ai, Yizhuang Yang, Yue Zhao, Liyan Wang, Juan Wang

**Affiliations:** 1 Department of Gastroenterology, The First Affiliated Hospital of Guilin Medical University, Guilin, China; 2 Department of Gastroenterology, Yueyang Central Hospital, Yueyang, China; 3 Guangxi Clinical Research Center for Early Diagnosis and Treatment of Gastric Cancer, Guilin, China; 4 Department of Pain Treatment, PKUCare Luzhong Hospital, Zibo, China; 5 Department of Pharmacy, Guilin Medical University, Guilin, China; 6 Faculty of Basic Medicine, Guilin Medical University, Guilin, China; 7 College of Nursing, Guilin Medical University, Guilin, China

**Keywords:** cell proliferation, ferroptosis, glycolysis, hepatocellular carcinoma (HCC), ubiquitination

## Abstract

Acidic leucine-rich nuclear phosphoprotein 32A(ANP32A) is known to possess multi-functions and has been observed to exhibit different roles across various malignancies. Although the expression of ANP32A has been shown to correlate with metastatic spread and reduced survival in Hepatocellular carcinoma(HCC), the underlying mechanisms have not been fully elucidated. This study utilized univariate Cox regression, differential gene analysis, and WGCNA to identify ANP32A as closely associated with poor HCC prognosis. Our Co-IP results confirmed the interaction between ANP32A and LDHA. Additionally, silencing ANP32A inhibited HCC cell proliferation *in vitro* as well as *in vivo*, and altered glycolytic and ferroptotic pathways, with increased LDHA ubiquitination detected. These data suggested that ANP32A may play a role in HCC progression, positioning it as a highly attractive therapeutic target for advanced HCC. Silencing ANP32A augmented the susceptibility of HCC cells to ferroptosis and inhibited aerobic glycolysis, which may provide new avenues for combination therapies, offering potential for improved patient outcomes.

## 1 Introduction

For HCC ranks among the world’s most prevalent cancers and is the third leading cause of cancer mortality (1–3). The early progression of HCC is typically insidious, with a lack of evident pathological symptoms. As a result, it is common for most patients to be diagnosed at an advanced stage of the disease. In the early phases of liver cancer, surgical resection is the preferred therapeutic option (4–6). Sorafenib, lenvatinib, and regorafenib have shown the ability to improve survival rates and are recognized as standard therapies for managing advanced HCC (7, 8). However, there is no definitive curative treatment for advanced HCC at present. Moreover, tumor recurrence and metastasis usually caused the treatment failure, leading to the poor survival for HCC patients. Thus, elucidating HCC’s molecular mechanisms and identifying therapeutic targets is imperative.

The Warburg effect, first described by Otto Warburg in the 1920s, is a hallmark of cancer metabolism and plays a crucial role in HCC progression (9, 10). It refers to the phenomenon where tumor cells, Although oxygen is present, mitochondrial oxidative phosphorylation is not utilized for energy production, instead, aerobic glycolysis supplies the energy required for their malignant proliferation (11, 12). This alteration in metabolism provides the energetic support cancer cells require as they undergo malignant growth. Increasing studies have shown that glycolysis and ferroptosis may exhibit a complex interplay (13–15), which is a category of cell death that was recently established to be dependent on iron (15, 16). This process is featured by the buildup of lipid peroxides along with iron ions (17, 18). Glycolysis occurs accompanied by the inhibition of ferroptosis in liver fibrosis. LDHA, serves as a critical factor that drives the Warburg effect, is reported negatively regulate ferroptosis induction in colorectal cancer treatment (19–21). However, it remains unclear the effect of LDHA-mediated glycolysis on ferroptosis induction in HCC and its role in the prognosis of HCC.

ANP32A, also referred to as PP32I-1, PP2A, Lanp, and PHAPI, is a multifunctional protein belonging to the ANP family (22), which is relatively conserved throughout evolution. It mediates several key cellular processes, from differentiation and apoptosis to cell cycle advancement (23, 24). Initially, ANP32A was considered a tumor suppressor (25, 26). Subsequent studies, however, have linked ANP32A to poor prognosis in several cancers, including colon cancer, leukemia, gastric cancer, and squamous cell carcinoma (27–30). Research has shown that ANP32A enhances the invasive and migratory abilities of HCC (31, 32). Recently, we found that ANP32A can interact with LDHA using IP analysis in SK-HEP-1 cells, which indicated that ANP32A may be involved in the glycolysis process of HCC. Meanwhile, our study showed that silencing ANP32A shortened the half-life of LDHA. However, the influence of the interaction between ANP32A and LDHA on the proliferation of HCC and whether ANP32A regulates glycolysis is still unclear.

In this study, we initially employed public databases to explore that a significant correlation exists between ANP32A and unfavorable prognosis in HCC. Then, we aim to investigate that ANP32A interacted with LDHA and regulated the ubiquitination degradation of LDHA, thereby influencing ferroptosis in HCC, suggesting that targeting ANP32A might be a potential treatment approach.

## 2 Materials and methods

### 2.1 Cell culture and cell transfection

In DMEM (Gibco) enriched with 10% fetal bovine serum (FBS, Clark Bioscience, USA) and 1% penicillin-streptomycin(Solarbio, Beijing, China), SK-HEP-1, Huh-7, and 293T cells were cultured under standard conditions (37 °C, 5% CO_2_) in an Eppendorf C170 incubator. For transfection, 293T cells were cultured in six-well plates and maintained until they reached 50% confluence before viral packaging. siRNA targeting ANP32A (50 nM) was obtained from General Biol and transfected with Lipofectamine 3000 reagent (Thermo Fisher Scientific). The sequences of ANP32A siRNA were 5′-CAAUCGCAAACUUACCAAAGTT-3′ and 5′-CUUUGGUAAGUUUGCGAUUGTT-3′. The sequences of negative control siRNA (si-NC) were 5′-UUCUCCGAACGUGUCACGUTT-3′ and 5′-ACGUGACACGUUCGGAGAATT-3′. Additionally, ANP32A overexpression (OE-ANP32A) and knockdown (sh-ANP32A) plasmids were obtained from Genechem (Shanghai), with con238 and con077 plasmids serving as controls for overexpression and knockdown experiments, respectively. Stable transfectants were selected by puromycin (5 μg/ml, Solarbio, Beijing, China). Green fluorescence was used to monitor successful infection.

### 2.2 TCGA, GEO and GTEx data source, processing and differential analysis

RNA sequencing raw data, along with the corresponding clinical details of liver HCC, were retrieved from TCGA online database: https://tcga-data.nci.nih.gov/tcga/. GEO data were retrieved from https://www.ncbi.nlm.nih.gov/geo/. GTEx data were retrieved from https://gtexportal.org/home/, DGE analysis was conducted by comparing tumor tissues using the limma R package (|LogFC| > 1, adjusted *P*-value (FDR) < 0.05). The resulting lists of upregulated genes were compiled for further analysis.

The TCGA-LIHC dataset consisted of 374 HCC tumor samples together with 50 matched adjacent non-tumor tissues. The GSE45436 series from the GEO repository served as an independent validation cohort, and normal hepatic transcriptomic data were retrieved from the GTEx database. All expression matrices were processed with log2 transformation and quantile normalization. The ComBat function embedded in the sva R package was used to eliminate cross-platform batch effects during the integration of TCGA and GTEx data. The Benjamini–Hochberg false discovery rate correction was implemented for all P values derived from univariate Cox regression to reduce false positive signals. The survminer R package was utilized to determine the optimal expression cutoff for stratifying patients based on ANP32A levels. Weighted gene co-expression network analysis (WGCNA) was performed on TCGA transcriptomic profiles, followed by module-trait correlation analysis to screen gene modules associated with HCC clinical outcomes for subsequent hub gene identification.

### 2.3 Cell viability assay

Logarithmic-phase Huh-7 and SK-HEP-1 HCC cells were transferred to 96-well plates at 4 × 10³ cells per culture well. Cells were co-treated for 48 h with 10 mM 2-DG (a glycolysis inhibitor, Aladdin, China) and 10 μM Erastin (a ferroptosis inducer, Aladdin, China). Then, MTT(Solarbio, Beijing, China) was introduced to the cells, followed by a 4-hour incubation to permit formazan crystal formation. Subsequently, Each well received 150 μL of DMSO(Solarbio, Beijing, China), and a TECAN Infinite M200 Pro NanoQuant microplate reader (Männedorf, Switzerland) was utilized to measure absorbance at 490 nm.

### 2.4 Reactive oxygen species assay

Transfected cells were incubated in 6 cm dishes until reaching approximately 60% confluence, after that they were treated with either a glycolysis inhibitor or a ferroptosis activator. Following treatment, cells were collected via digestion with 0.25% trypsin (Sigma-Aldrich). After centrifugation and removal of the supernatant, 0.3 mL of PBS(Abbkine) containing 2 mM of the ROS probe DHE (Beyotime Biotechnology, Shanghai, China) was applied, and the cells were allowed to stain for 20 minutes. Then, after rinsing, cells were resuspended in PBS and evaluated by flow cytometry using a BD Accuri C6 Plus Flow Cytometer.FlowJo(v10.8) software was utilized for data analysis.

### 2.5 Mitochondrial membrane potential detection

It was assessed using a procedure similar to that used for ROS detection. After centrifugation, a volume of 0.3 mL of PBS supplemented with 0.2 μM JC-1 fluorescent probe(Beyotime Biotechnology, Shanghai, China) was utilized to resuspend the cells. Cells were stored in darkness for 20 minutes during incubation. Subsequently, PBS served as the rinsing agent for the cells, and alterations in mitochondrial membrane potential (MMP) were assessed by flow cytometry. The mean fluorescence intensity (MFI) was determined with FlowJo software.

### 2.6 Western blot analysis

RIPA buffer(Solarbio, Beijing, China) was applied to disrupt the cell pellets for at least 30 minutes, the protein was separated by centrifugation. After detected the protein concentrations, and the samples were prepared with SDS(Solarbio, Beijing, China) loading buffer. Western blot analysis was performed as previously reported (33). Primary antibodies were Ub (WanleiBio #WL01368, 1:1000), GLUT1 (abcam #ab115730, 1:5000), TFR1 (ZenBio #381603, 1:1000), COX-2 (EnoGene #E11-0160C, 1:1000), LDHA (Proteintech #19987-1-AP, 1:2000), GPX4 (Abmart #T56959, 1:1000), ANP32A (EnoGene #E2505768, 1:1000), FTH-1 (EnoGene #E1A21469C, 1:1000), HO-1 (EnoGene #E2382808, 1:2000), β-actin (Affinity #T0022, 1:5000), Flag (abcam #ab205606, 1:1000)and IgG (Abcam #ab172730, 1:1000).

### 2.7 Quantitative real-time PCR

The ABI-7500 system(Thermo Fisher Scientific, USA) was employed with the UltraSYBR One-Step RT-qPCR kit (CWBIO, Beijing). The primer sequences used for RT-qPCR are listed as follows:ANP32A: Forward: 5′-CACCTCAATCGCAAATCCATCCA-3′, Reverse: 5′-AACACATTTTCTCGGTAGTCGTT-3′; LDHA: Forward: 5′-CATTGAAGCAAACAATAAGTAGAGG-3′, Reverse: 5′-TGGAGCACATAGCTCAGGTTT-3′; GLUT1 Forward: 5′-CAGTCGCCTGATAACACTGTG-3′, Reverse: 5′-GCCCGCGAGAGGAAGATG-3′; PKM2:Forward: 5′-GCCGCCTGACATTGACTC-3′, Reverse: 5′-CCATGGAGAGAATTCGGCAG-3′; HK2:Forward: 5′-TGATCGCCTGCTTATTCACGG-3′, Reverse: 5′-AACCGGCTGAGAATTCGCAGA-3′; β-Actin: Forward: 5′-CATGTACGTTGCTATCCAGGC-3′ Reverse: 5′-CTCCTTAATGTCACGCACGAT-3′. The reaction was prepared in accordance with the manufacturer’s instructions, and the thermal cycling conditions included reverse transcription followed by PCR amplification. The comparative Ct (ΔΔCt) method served to determine relative gene expression.

### 2.8 GO and KEGG enrichment analysis

The predicted ANP32A-interacting genes were analyzed for Biological activities and signaling pathways using the enrichment analyses of GO and KEGG. GO analysis identified significantly enriched signaling pathways, highlighting genes involved in specific biological functions. KEGG analysis further elucidated functional pathways, with significance set at *P* < 0.05.

### 2.9 Co-immunoprecipitation assay

Lysis buffer containing protease inhibitor cocktail(Solarbio, Beijing, China) at a 1:100 dilution, PMSF(1:100, Solarbio, Beijing, China), and 1 μL/mL DL-dithiothreitol (DTT, Solarbio, Beijing, China), was used to lyse cells. After incubating cell lysates with the target protein antibody, the immune complexes were captured by adding Protein A/G magnetic beads (MedChemExpress, HY-K0202). To eliminate non-specifically bound proteins, IP buffer(Pierce, Thermo Fisher Scientific, USA) was used to wash the beads. Captured proteins were eluted, heated, and subsequently analyzed by Western blot.

### 2.10 Immunofluorescence assay

Coverslips seeded with SK-HEP-1 and Huh-7 cells were fixed (4% paraformaldehyde, (Biosharp, Hefei, China)), permeabilized (0.01% Triton X-100), blocked (5% BSA), and exposed to anti-ANP32A(EnoGene #E2505768, 1:200) and anti-LDHA (Proteintech #19987-1-AP, 1:300) primary antibodies. Red and green fluorescent secondary antibodies (1:500) were used to detect ANP32A and LDHA, respectively. PBS was used for washing the cells thrice between each step. After staining the nuclei with DAPI (Beyotime, Shanghai), fluorescence images were taken on a confocal laser scanning microscope (Nikon, Japan) to evaluate the localization and levels of ANP32A and LDHA.

### 2.11 Glucose uptake and lactate production

ANP32A knockdown and overexpression groups were placed in 6-well plates and cultured with the glycolysis inhibitor 2-deoxyglucose (2-DG) at 10 mM final concentration. After incubation for 48 hours, both the cells and the culture fluid were collected. A Glucose Detection Kit from Applygen (Beijing) was utilized to calculate glucose uptake following the manufacturer’s protocol, by assessing medium glucose levels before and after treatment. A Lactate Assay Kit (Jiancheng Bioengineering, Nanjing) was used to quantify lactate levels in line with the manufacturer’s protocol. Data were adjusted based on the cell number per well to compensate for differences in cell density.

### 2.12 Iron assay

Knockdown or overexpression of ANP32A cells were plated and exposed to 10 mM glycolysis inhibitor 2-deoxyglucose (2-DG) or 10 μM ferroptosis activator Erastin. To acquire the cell pellet, cells were collected by centrifugation after 48 hours of incubation. A Total Iron Colorimetric Assay Kit (Applygen, Beijing) was employed to quantify the total intracellular iron concentration. The assay involved the colorimetric detection of iron levels in the cell lysates, and normalization was performed using the cell count per sample to compensate for differences in cell density.

### 2.13 Malondialdehyde assay and superoxide dismutase assay

RIPA buffer(Solarbio, Beijing, China) was used for cell disrupt after the treated cells had been rinsed with PBS. The manufacturer’s instructions were followed, MDA levels were evaluated with an MDA Assay Kit (TBA method, Jiancheng Bioengineering Institute, Nanjing), and SOD activity was determined using a SOD Assay Kit (WST-1 method, Jiancheng Bioengineering Institute, Nanjing). MDA was detected by its reaction with thiobarbituric acid (TBA), and SOD activity was assessed by its inhibition of WST-1 reduction. Results were normalized to total protein concentration.

### 2.14 Protein half-life assay

To evaluate the LDHA protein half-life, the translation inhibitor cycloheximide (CHX, Solarbio, Beijing, China) was added to SK-hep-1 and Huh-7 cells at 10 μg/mL and then collected at specific time intervals. After sample collection, LDHA protein expression was analyzed via Western blot to monitor the degradation rate over time, allowing for the determination of its half-life.

### 2.15 Xenograft mouse model

The animal study protocol was permitted by the Institutional Animal Care and Use Committee of Guilin Medical University (Guilin, China) (protocol code GLMC-IACUC-20241101). Male BALB/c nude mice (five weeks of age) were sourced from Hunan SJA Laboratory Animal Co., Ltd. (Hunan, China)(Qualification Certificate No: (Xiang) 2019-0004) and were acclimated for one week in an SPF environment. Mice were randomly assigned to two groups using a random number table before cell injection. An aggregate of 6 × 10^6^ Huh-7 cells transduced with sh-ANP32A or sh-NC were injected subcutaneously into nude mice. Once the tumors formed, both tumor dimensions and body weight were evaluated every five days for a total of five weeks. Tumor length and width were measured by an investigator blinded to group information. Tumor volume was calculated with the formula: Volume = (length × width²)/2. No animals or tumor samples were excluded during the whole experiment. The experimental endpoint was set at five weeks after cell inoculation. Humane endpoint criteria were applied: mice would be euthanized immediately if they had more than 20% weight loss, severe tumor ulceration or necrosis, impaired movement and feeding, or apparent discomfort. Finally, mice were humanely killed under profound anesthesia induced via intraperitoneal injection dose of pentobarbital sodium (40 mg/kg). Xenograft tumors were dissected on ice and separated from adjacent skin and adipose tissue, followed by rinsing with pre-cooled PBS to eliminate residual blood. Part of the neoplasm was immersed in 4% paraformaldehyde for fixation, while the remainder was snap-frozen in liquid nitrogen and subsequently stored at -80 °C for downstream experiments.

### 2.16 Statistics

All *in vitro* cell experiments were conducted with three independent biological replicates, and three technical replicates were set for each biological replicate. Independent biological replicates were arranged for *in vivo* xenograft experiments to support data reproducibility. SPSS (v22.0), R Studio (v4.2), and GraphPad Prism (v6) were analyzed statistically. Survival analysis was performed using the Kaplan–Meier method to assess the association between ANP32A expression and prognosis in HCC patients. One-way ANOVA compared differences among groups, while the connection between gene expression and survival outcomes was further evaluated using Cox regression analysis. *P* < 0.05 was used as the criterion for statistical significance.

## 3 Results

### 3.1 Elevated ANP32A levels were associated with a poor prognosis in HCC

**Figure 1:**
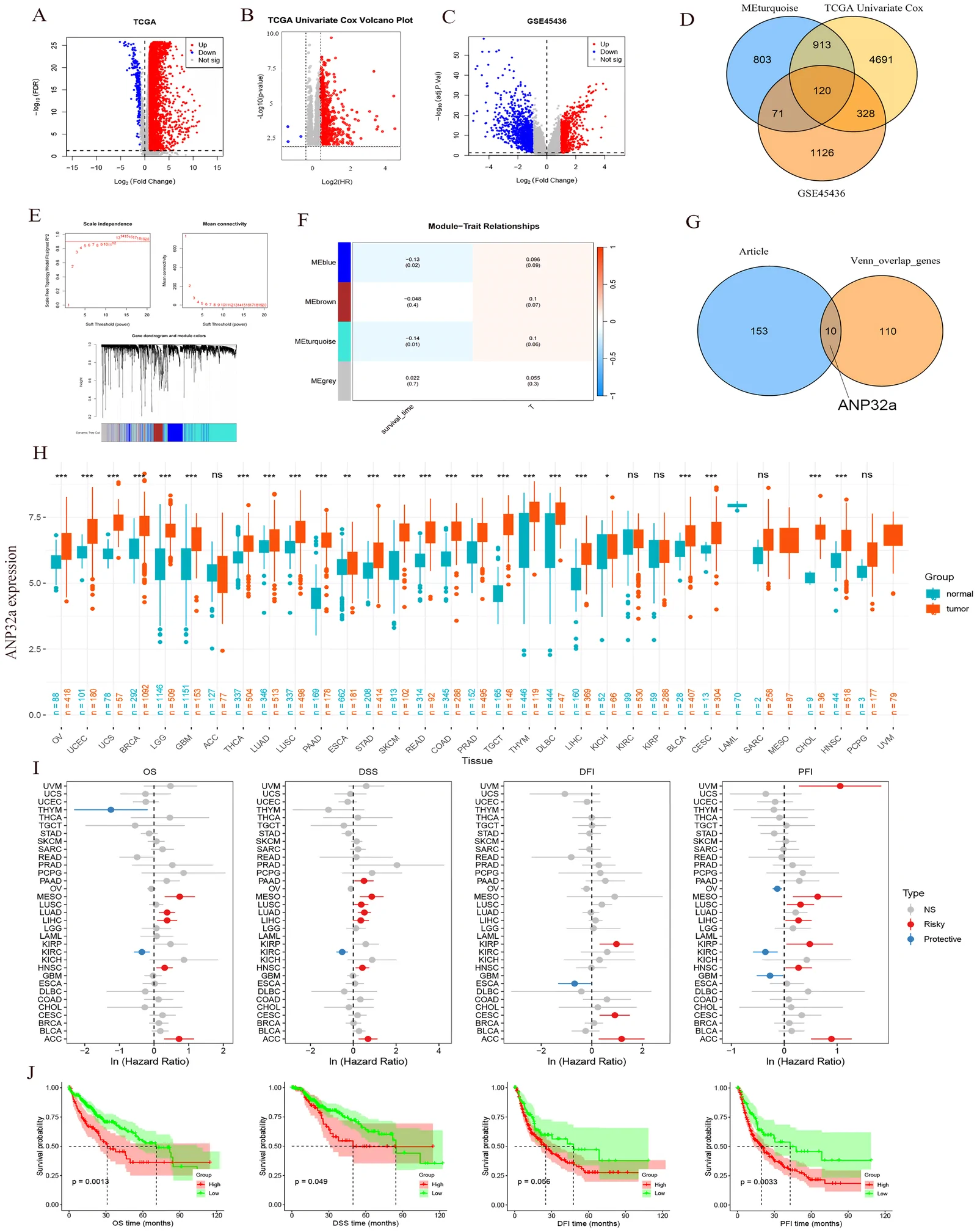
Integrated screening identifies ANP32A as a pivotal regulator of survival in HCC patients. **(A)** DEG was carried out using TCGA gene expression data, comparing liver cancer with normal tissues, and visualized with a volcano plot. **(B)** Univariate Cox analysis of TCGA upregulated genes assessed their relationship with survival time, displayed in a volcano plot. **(C)** DEG analysis was performed on the GSE45436 dataset with a volcano plot. **(D)** A Venn diagram was created using TCGA univariate Cox genes, GSE45436 DEGs, and the MEturquoise module. **(E)** WGCNA analysis of TCGA data identified four modules associated with survival time and T stage. **(F)** Module–trait correlation heatmap. **(G)** Venn diagram displaying ten overlapping genes from two gene sets, with ANP32A further screened as the target gene. **(H)** Pan-cancer differential analyses of ANP32A were performed using GTEx and TCGA data. **(I)** Pan-cancer univariate Cox forest plots illustrating the prognostic associations of ANP32A with OS, DSS, DFI and PFI. **(J)** Kaplan-Meier survival curves analyzed ANP32A’s correlation with these metrics in LIHC patients. ***P < 0.001, ns, P > 0.05, vs normal group.

Firstly, we performed DEG analysis using the TCGA database and selected upregulated genes for univariate Cox analysis. This analysis identified 6,052 genes significantly associated with HCC prognosis (**Figures 1A, B**). Thus, we obtained the HCC GEO dataset (GSE45436) and performed DEG analysis to compare tumor tissues against adjacent normal tissues. The DEGs were visualized using Volcano Plots (**Figure 1C**). Second, we identified 4 WGCNA modules related to survival time and tumor stage of HCC (**Figure 1E**) and followed by the evaluation of module-trait analysis (**Figure 1F**). Although multiple modules were associated with HCC survival time to varying degrees, the MEturquoise module showed a significant correlation while having the lowest absolute r value (r = -0.14) (**Figure 1F**). Using the aforementioned methods, we identified 120 genes associated with HCC prognosis (**Figure 1D**). To further investigate, we conducted a literature review of HCC proteomics studies and intersecting these 120 prognostic genes with previously reported HCC-related molecules from published proteomics research to obtain ten overlapping genes (**Figure 1G**). ANP32A was selected for subsequent functional validation based on its clinical relevance and biological characteristics, given that its potential involvement in metabolic reprogramming, especially glycolysis and ferroptosis, remains largely uncharacterized in HCC.

Subsequently, we conducted pan-cancer differential expression analysis and univariate Cox regression analysis of ANP32A using the combined GTEx and TCGA databases (**Figures 1H, I**). Overall survival (OS), disease-specific survival (DSS), and progression-free interval (PFI) were all significantly associated with ANP32A expression in liver cancer patients, while no significant association was detected for disease-free interval (DFI)(**Figure 1I**). Kaplan-Meier survival analysis and log-rank tests were applied to assess the relationship between ANP32A expression and OS, DSS, DFI, and PFI in HCC patients. High ANP32A expressers showed significantly worse OS, DSS, and PFI than those with diminished expression, according to the analysis (**Figure 1J**). In summary, ANP32A expression was a prognostic marker for HCC patients.

### 3.2 ANP32A promotes glycolysis and enhances proliferation in HCC cells

**Figure 2:**
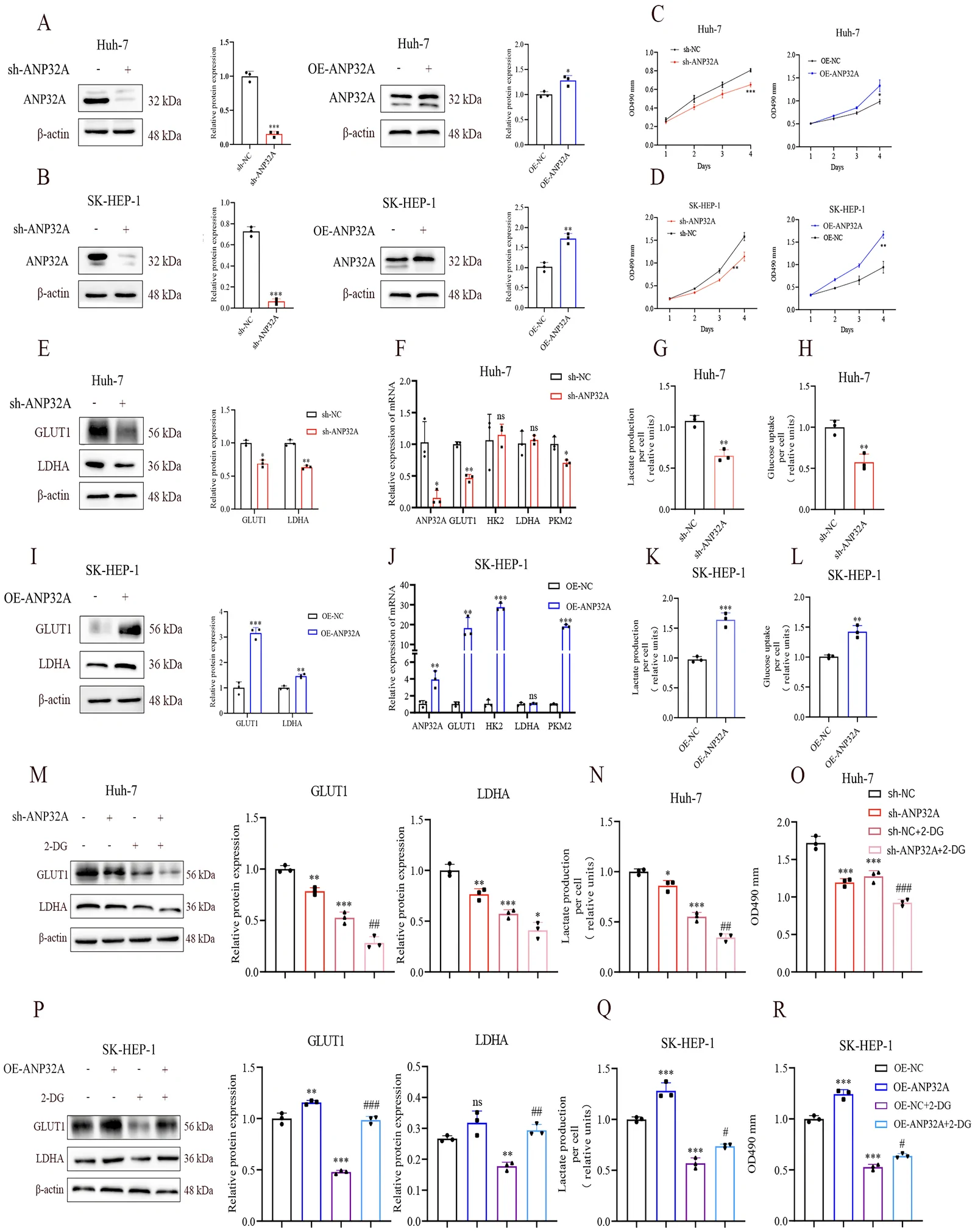
Silencing ANP32A inhibits HCC cell proliferation and suppresses glycolysis. **(A, B)** Western blot was employed to detect the expression levels of ANP32A in Huh-7 and SK-HEP-1 cells. **(C, D)** MTT assays assessed the effects of ANP32A deficiency or overexpression on the expansion of Huh-7 and SK-HEP-1 cells. **(E, I)** Immunoblot analysis of LDHA and GLUT1 in Huh-7 and SK-HEP-1 cells with indicated transfection. **(F, J)** RT-qPCR was employed to detect the expression levels of glycolysis-related factor mRNA. **(G, H, K, L)** Glucose uptake and lactate production were measured when ANP32A was knocked down in Huh-7 cells and overexpressed in SK-HEP-1 cells. **(M, P)** Western blot was employed to detect the protein expression of LDHA and GLUT1 after the addition of 2-DG to the transfected cell lines. **(N, Q)** Lactate production was measured after adding 2-DG to the transfected cell lines. **(O, R)** MTT was used to assess cell proliferation after adding 2-DG to the transfected cell lines. The internal control was β-actin. The above values are expressed as mean ± SD, n = 3, *P < 0.05, **P < 0.01, ***P < 0.001, ns, *P* > 0.05, *vs* sh-NC or OE-NC; ^#^*P* < 0.05, ^##^*P* < 0.01, ^###^*P* < 0.001, ns, *P >*0.05 *vs* sh-ANP32A+2-DG or OE-ANP32A+2-DG.

In this work, we used the ANP32A-knockdown and ANP32A-overexpressing cells to explore its potential impact on glucose metabolism in HCC. After transfection, ANP32A expression showed significant changes at both the RNA and protein levels (**Figures 2A, B, F, J**). MTT assays showed that ANP32A downregulation inhibited liver cancer cells proliferation, whereas upregulation promoted it (**Figures 2C, D**).

Our findings showed that ANP32A knockdown decreased the protein levels of GLUT1 and LDHA (**Figure 2E** and **Supplementary Figure 1A**), while also decreasing the mRNA levels of GLUT1, PKM2, and HK2, without affecting the mRNA levels of LDHA (**Figure 2F** and **Supplementary Figure 1B**), while overexpressed ANP32A resulted in opposite changes on these factors (**Figure 2I, J** and **Supplementary Figures 1E, F**). Notably, GLUT1 and LDHA were validated at both mRNA and protein levels, whereas HK2 and PKM2 were only detected at the mRNA level in the current study. Furthermore, cellular glucose uptake and lactate production were assessed, and ANP32A deficiency was found to reduce both(**Figures 2G, H** and **Supplementary Figures 1C, D**). Conversely, ANP32A overexpression promoted glucose uptake and lactate production (**Figure 2K, L** and **Supplementary Figures 1G, H**).

To further validate ANP32A’s impact on glycolysis, we used the glycolysis inhibitor 2-DG. 2-DG exacerbated the reduction of LDHA and GLUT1 protein levels caused by ANP32A knockdown (**Figure 2M** and **Supplementary Figure 1I**) and suppressed their upregulation caused by ANP32A overexpression (**Figure 2P** and **Supplementary Figure 1L**). It also enhanced the lactate decrease in shANP32A cells (**Figure 2N** and **Supplementary Figure 1J**) and mitigated the increase in ANP32A overexpressed cells (**Figure 2Q** and **Supplementary Figure 1M**). MTT analysis indicated that 2-DG suppressed the proliferation of liver cancer cells, reversed the increased cell viability induced by ANP32A overexpression in SK-HEP-1 and Huh-7 cells (**Figure 2O, R** and **Supplementary Figure 1K, N**). In conclusion, the present study showed that ANP32A positively regulated cancer cell proliferation, glucose uptake and lactate production by modulating the expression of glycolysis-related genes in HCC cells.

### 3.3 ANP32A is correlated with altered LDHA ubiquitination in HCC cells

**Figure 3:**
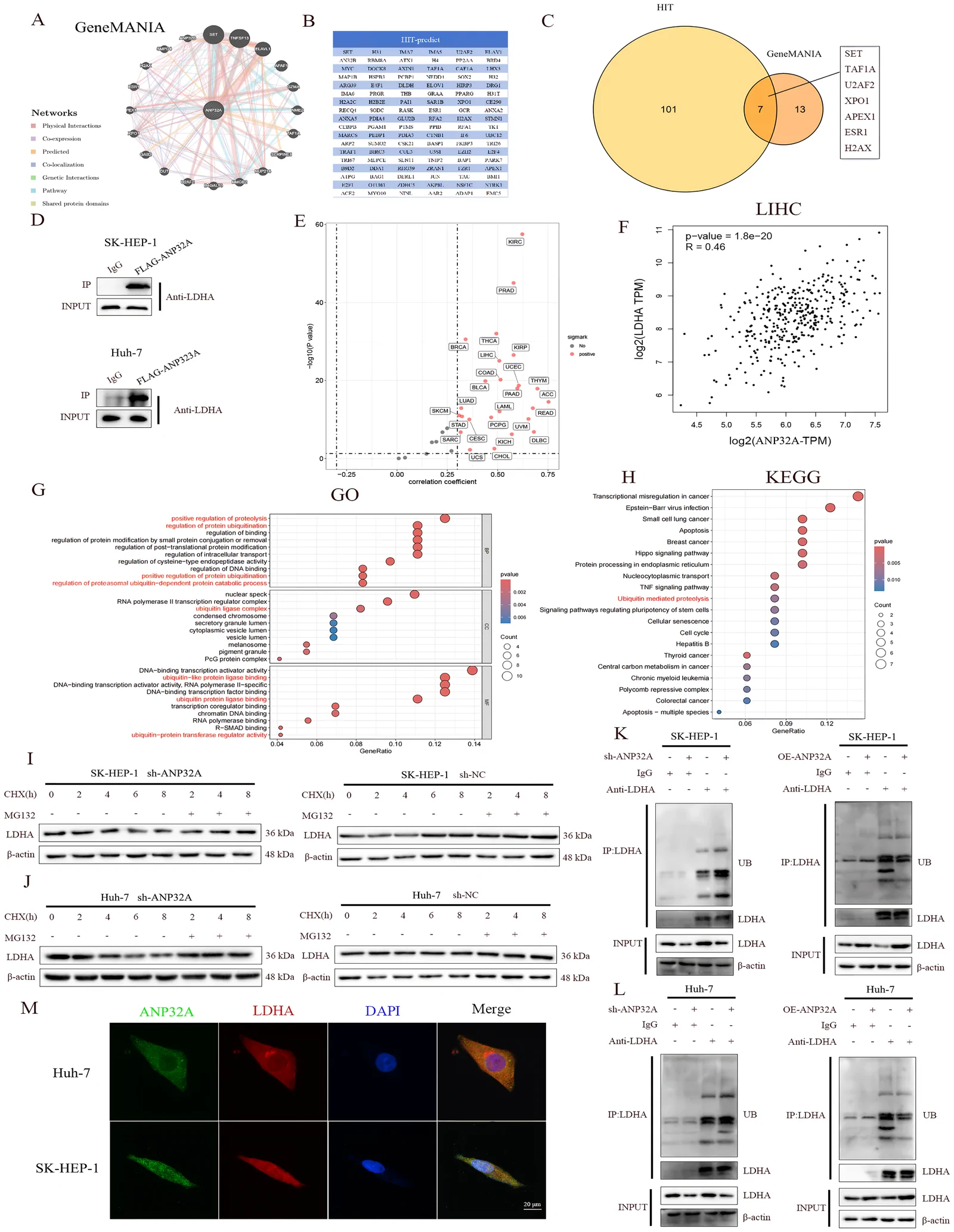
ANP32A activates LDHA by inhibiting its ubiquitination and degradation. **(A, B)** PPI prediction was conducted via GeneMANIA and HIT-prediction. **(C)** A Venn diagram was employed to intersect the genes identified by the two prediction methods. **(D)** The interaction analysis between ANP32A and LDHA was assessed by Co-IP. **(E)** A pan**-**cancer correlation analysis of ANP32A and LDHA was performed using TCGA data. **(F)** A scatter plot was used to illustrate the expression levels of ANP32A and LDHA in LIHC patients. **(G, H)** GO and KEGG pathway enrichment analysis based on proteins predicted to interact with ANP32A. **(I, J)** Western blot was utilized to examine the half-life of LDHA in SK-HEP-1 and Huh-7 cells with ANP32A knockdown and control groups, following the addition of CHX and MG132 at regular time intervals. **(K, L)** Western blot analysis was performed after collecting transfected SK-HEP-1 and Huh-7 cells for a Co-IP experiment. **(M)** Immunofluorescence images of ANP32A (green) and LDHA (red) staining in SK-HEP-1 and Huh-7 cells. Nuclei were visualized with DAPI (blue). Scale bar: 20 μm.

To elucidate the mechanistic basis of ANP32A-mediated glycolytic regulation in HCC, we performed in silico PPI network analysis with GeneMANIA (34)and HIT-prediction (10) to predict candidate substrates(**Figures 3A, B**). Since the predicted overlapping genes showed no significant correlation with glycolysis(**Figure 3C**), we performed a Co-IP together with proteomics to identify the target proteins interacting with ANP32A. LDHA was screened out from proteomic results, and interestingly, we found that LDHA was one of the specific interacting proteins with ANP32A (**Figure 3D**). A positive correlation between ANP32A and LDHA expression was observed across multiple cancers in the TCGA database (**Figure 3E**), with this correlation being especially pronounced in liver cancer (**Figure 3F**).

We have found that ANP32A deficiency affects LDHA protein levels without impacting its mRNA expression (**Figures 2E, F, I, J**), suggesting that ANP32A may regulate the post-translational modification of LDHA. To identify the type of modification, GO and KEGG enrichment analyses were performed on a set of 121 genes predicted to interact with ANP32A. Both two analyses method all enrichment in ubiquitination-related pathways (**Figures 3G, H**). Furthermore, we treated ANP32A knockdown and control cells with cycloheximide (CHX), a protein synthesis inhibitor, to determine the half-life of LDHA. Additionally, we used the proteasome inhibitor MG132 (carbobenzoxy-Leu-Leu-leucinal) to inhibit the proteasomal degradation pathway within the cells. After 4 hours of CHX treatment, ANP32A knockdown led to a significant decrease in LDHA levels. Consistently, the cells with ANP32A knockdown showed accelerated LDHA degradation relative to the control group. Collectively, these findings indicate that ANP32A knockdown resulted in a shortened LDHA half-life in SK-HEP-1 and Huh-7 cells, and this effect was suppressed by MG132(**Figures 3I, J**). Co-IP assays showed that ANP32A knockdown increased the ubiquitination level of LDHA, while ANP32A overexpression reduced LDHA ubiquitination (**Figures 3K, L**). Confocal imaging showed the co-localization of ANP32A (green) and LDHA (red) in the cytoplasm of SK-HEP-1 and Huh-7 cells(**Figure 3M**). In conclusion, ANP32A interacts with LDHA and correlates with decreased ubiquitination and degradation of LDHA, which may affect glycolysis in HCC cells.

### 3.4 ANP32A regulates ferroptosis in HCC cells

**Figure 4:**
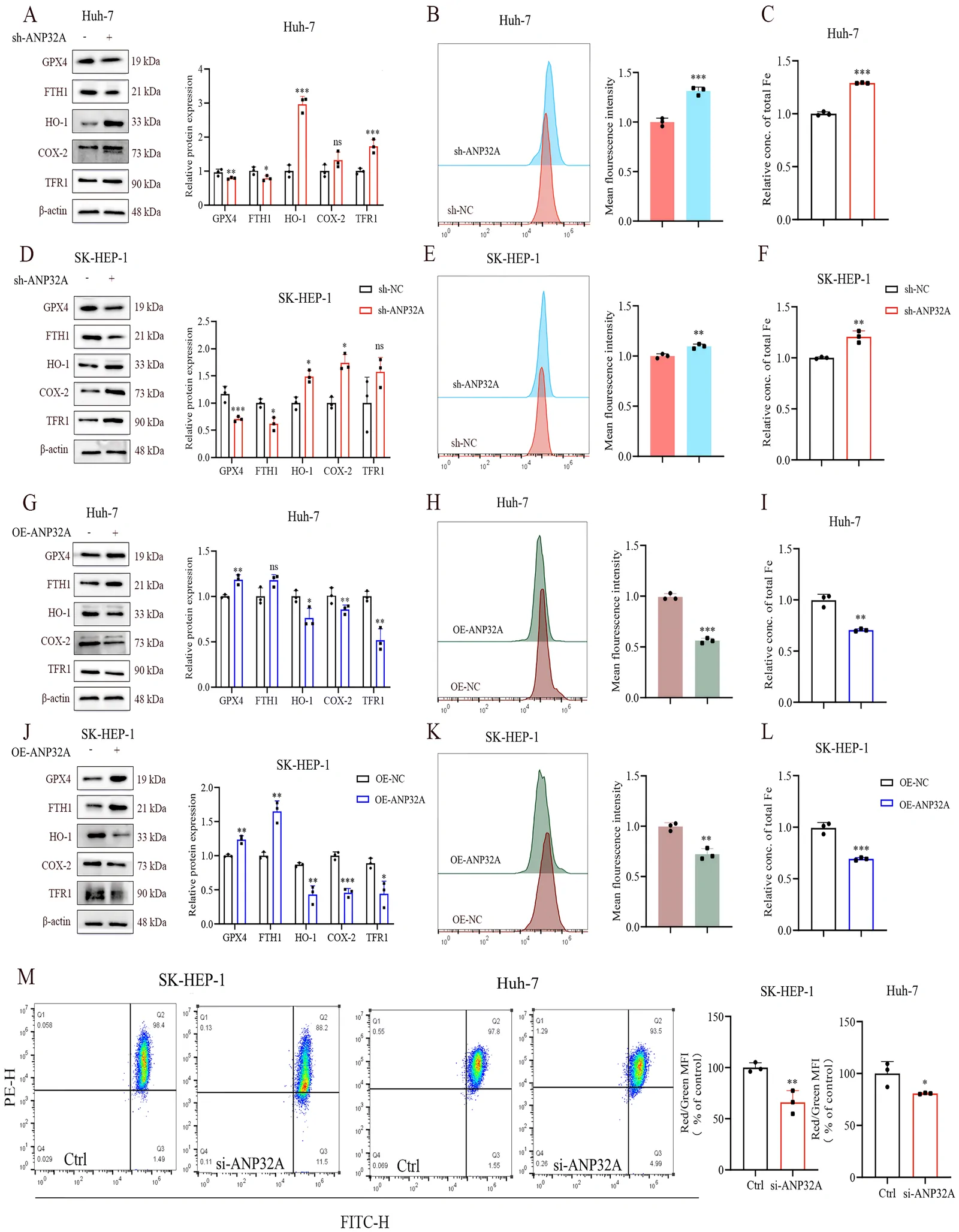
ANP32A modulates ferroptosis in HCC cells. **(A, D)** Western blot analysis of ferroptosis-related gene proteins following ANP32A knockdown. **(B, E)** Flow cytometry was performed to detect intracellular ROS after ANP32A silencing. **(C, F)** Total iron assay kit served to quantify intracellular total iron following ANP32A silencing. **(G, J)** Western blot analysis of ferroptosis-related gene proteins following ANP32A overexpression. **(H, K)** Flow cytometry was performed to detect intracellular ROS after ANP32A overexpression. **(I, L)** Total iron assay of intracellular iron concentration after ANP32A overexpression. **(M)** JC-1 staining served to evaluate changes in mitochondrial membrane potential induced by siRNA-mediated transient ANP32A knockdown. The above values are expressed as mean ± SD, n = 3, *P < 0.05, **P < 0.01, ***P < 0.001, ns, *P* > 0.05, *vs.* sh-NC or OE-NC.

Western Blot analysis indicated that ANP32A downregulation reduced the expression of key ferroptosis-associated proteins GPX4 and FTH-1, while elevating those of HO-1, COX-2, and the iron transport protein TFR1. In contrast, opposite changes on the above ferroptosis-related proteins were observed in the cells overexpressed ANP32A (**Figures 4A, D, G, J**). We also found that ANP32A upregulation leading to decreased intracellular ROS levels (**Figures 4B, E, H, K**). Additionally, ANP32A depletion led to iron accumulation in SK-HEP-1 and Huh-7 cells, while ANP32A overexpression yielded the opposite effect(**Figures 4C, F, I, L**). Moreover, flow cytometry assays showed that a significant reduction in mitochondrial membrane potential was observed in ANP32A knockdown cells(**Figure 4M**). We used transient siRNA here to avoid lentiviral screening interference on mitochondria, whereas other experiments adopted stable shRNA cell lines. These findings strongly indicated that the expression of ANP32A was closely related to ferroptosis inhibition in HCC cells.

### 3.5 Knockdown of ANP32A enhances Erastin-induced ferroptosis

**Figure 5:**
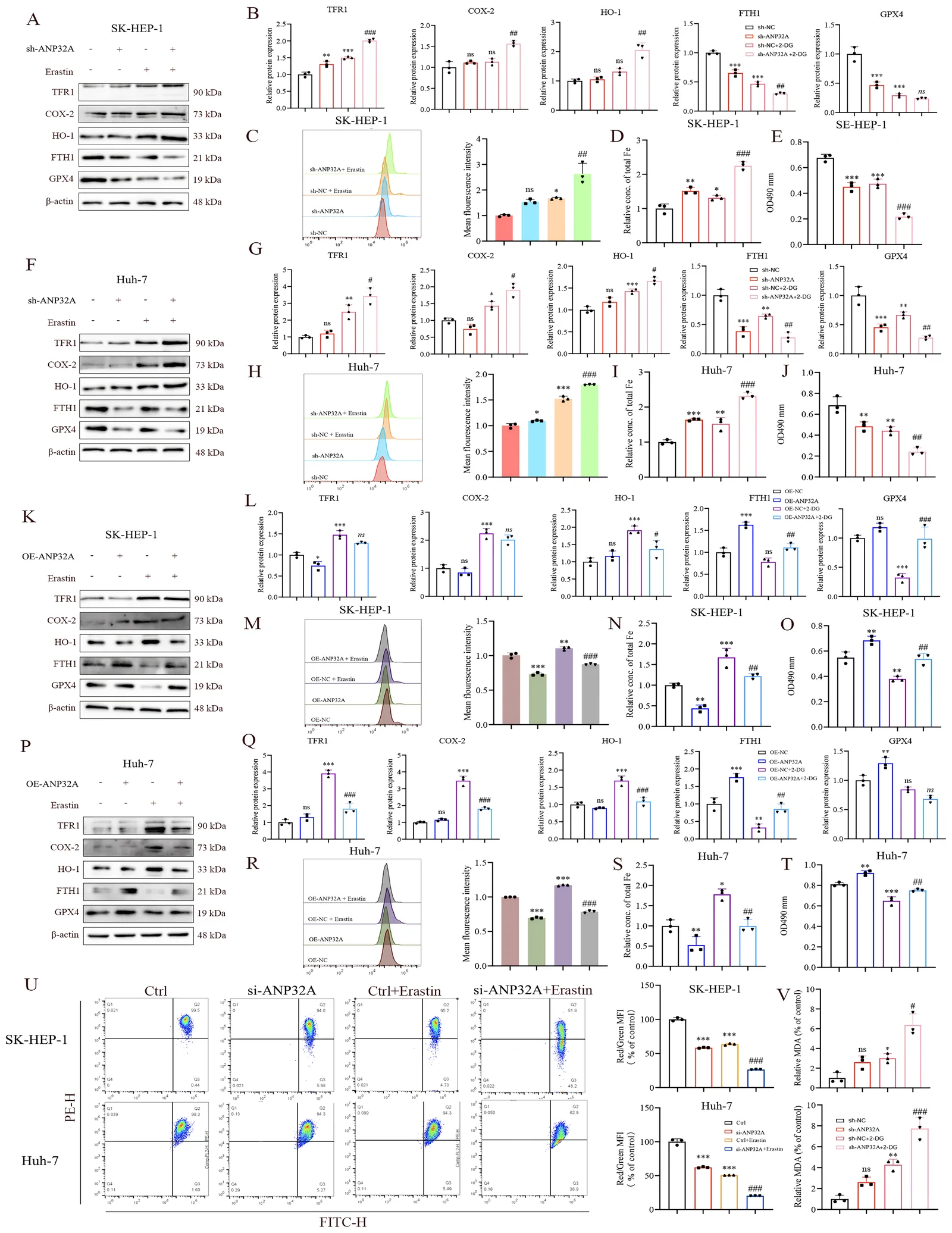
ANP32A depletion enhances Erastin-induced ferroptosis. **(A, B, F, G)** The expression levels of TFR1, COX-2, HO-1, FTH1, and GPX4 were analyzed by western blotting following Erastin treatment in cells with ANP32A silenced. **(C, H)** ROS production in HCC cell lines treated with Erastin in ANP32A silenced cells. **(D, I)** Total iron content was measured in ANP32A silenced cells following the treatment with Erastin. **(E, J)** Cell viability of the ANP32A silenced group treated with Erastin was assessed using the MTT assay. **(K, L, P, Q)** The expression levels of TFR1, COX-2, HO-1, FTH1, and GPX4 were analyzed by western blotting following Erastin treatment in cells with overexpressed ANP32A. **(M, R)** ROS production in HCC cell lines treated with Erastin in ANP32A overexpressed cells. **(N, S)** Total iron content was measured in ANP32A overexpressed cell lines following the treatment with Erastin. **(O, T)** MTT assay was performed to evaluate the viability of the ANP32A overexpressing cells after treated with Erastin. **(U)** The mitochondrial membrane potential of transient siRNA-mediated ANP32A-knockdown cells treated with Erastin was determined by flow cytometry. **(V)** MDA levels in Erastin-treated cells were assessed using an MDA assay kit. The above values are expressed as mean ± SD, n = 3, *P < 0.05, **P < 0.01, ***P < 0.001, ns, *P* > 0.05, *vs.* sh-NC or OE-NC; ^#^*P* < 0.05, ^##^*P* < 0.01, ^###^*P* < 0.001, ns, *P* > 0.05, *vs.* sh-ANP32A+Erastin or OE-ANP32A+Erastin.

The findings indicated that Erastin increased the expression of HO-1, COX-2, and TFR1 proteins in SK-HEP-1 and Huh-7 cells following ANP32A knockdown, while decreased GPX4 and FTH1 protein levels(**Figures 5A, B, F, G**). Moreover, Erastin reversed the impact of ANP32A overexpression on ferroptosis-related proteins(**Figures 5K, L, P, Q**). Flow cytometry assays revealed that the increased ROS level caused by ANP32A depletion, was further enhanced by Erastin(**Figures 5C, H**). Conversely, the reduction in ROS levels caused by ANP32A overexpression was inhibited by Erastin(**Figures 5M, R**). Additionally, we found that the Fe2+ concentration following Erastin treatment was increased(**Figures 5D, I, N, S**). MTT assay results revealed that Erastin intensified the proliferation inhibition resulting from ANP32A depletion (**Figures 5E, J**) and reversed the accelerated proliferation associated with ANP32A overexpression (**Figures 5O, T**). Moreover, MMP assay showed that ANP32A silencing exacerbated the Erastin-induced loss of MMP(**Figure 5U**).

After ANP32A knockdown, the intracellular MDA levels were significantly elevated, and this effect was further enhanced by the addition of Erastin (**Figure 5V**). Collectively, these results provided sufficient evidence that ferroptosis induced by ANP32A knockdown in HCC can be enhanced by Erastin, highlighting the negative regulation role of ANP32A in the ferroptosis process of these cells.

### 3.6 Glycolysis inhibition facilitates ferroptosis triggered by ANP32A depletion

To investigate the relationship between glycolysis and ferroptosis following ANP32A knockdown, we used glycolysis inhibitors 2-DG conducted the following experiments.

**Figure 6:**
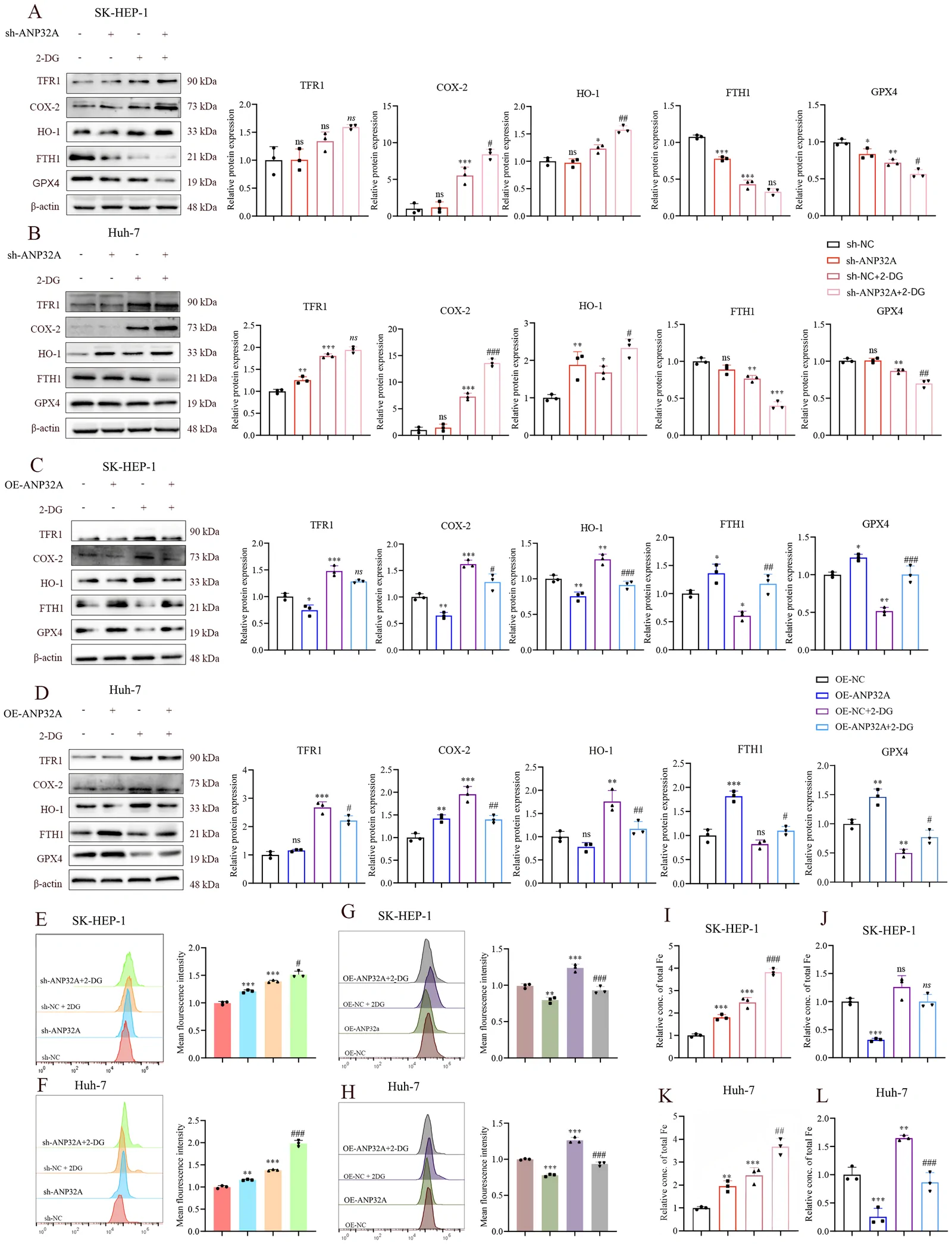
Glycolysis inhibition facilitates ferroptosis triggered by ANP32A depletion. **(A-D)** The expression levels of TFR1, COX-2, HO-1, FTH1, and GPX4 were analyzed by western blotting following treatment with 2-DG in ANP32A knockdown or overexpressing cells. **(E-H)** ROS production in HCC cell lines treated with 2-DG in ANP32A knockdown or overexpressed cells. **(I-L)** Total iron content was assessed in ANP32A knockdown or overexpressed cells after treatment with 2-DG. The above values are expressed as mean ± SD, n = 3, *P < 0.05, **P < 0.01, ***P < 0.001, ns, *P* > 0.05, *vs.* sh-NC or OE-NC; ^#^*P* < 0.05, ^##^*P* < 0.01, ^###^*P* < 0.001, ns *P* > 0.05, *vs.* sh-ANP32A + 2-DG or OE-ANP32A + 2-DG.

We found that 2-DG elevated HO-1, COX-2, and TFR1 protein levels in ANP32A knockdown HCC cells, while the GPX4 and FTH1 expression levels were decreased (**Figures 6A, B**), and the reverse effects were observed in HCC cells overexpressing ANP32A (**Figures 6C, D**). Additionally, intracellular ROS levels were also enhanced by 2-DG, similar to the effects observed in ANP32A knockdown cells (**Figures 6E, F**), whereas upregulation of ANP32A exerted the opposite effects(**Figures 6G, H**). After the addition of 2-DG, intracellular iron ion concentrations exhibited similar changes to those observed with Erastin treatment(**Figures 6I–L**). These results suggest that ANP32A knockdown inhibited glycolysis and promoted ferroptosis in HCC cells.

### 3.7 ANP32A depletion suppressed cell proliferation and glycolysis as well as inducing ferroptosis *in vivo*

**Figure 7:**
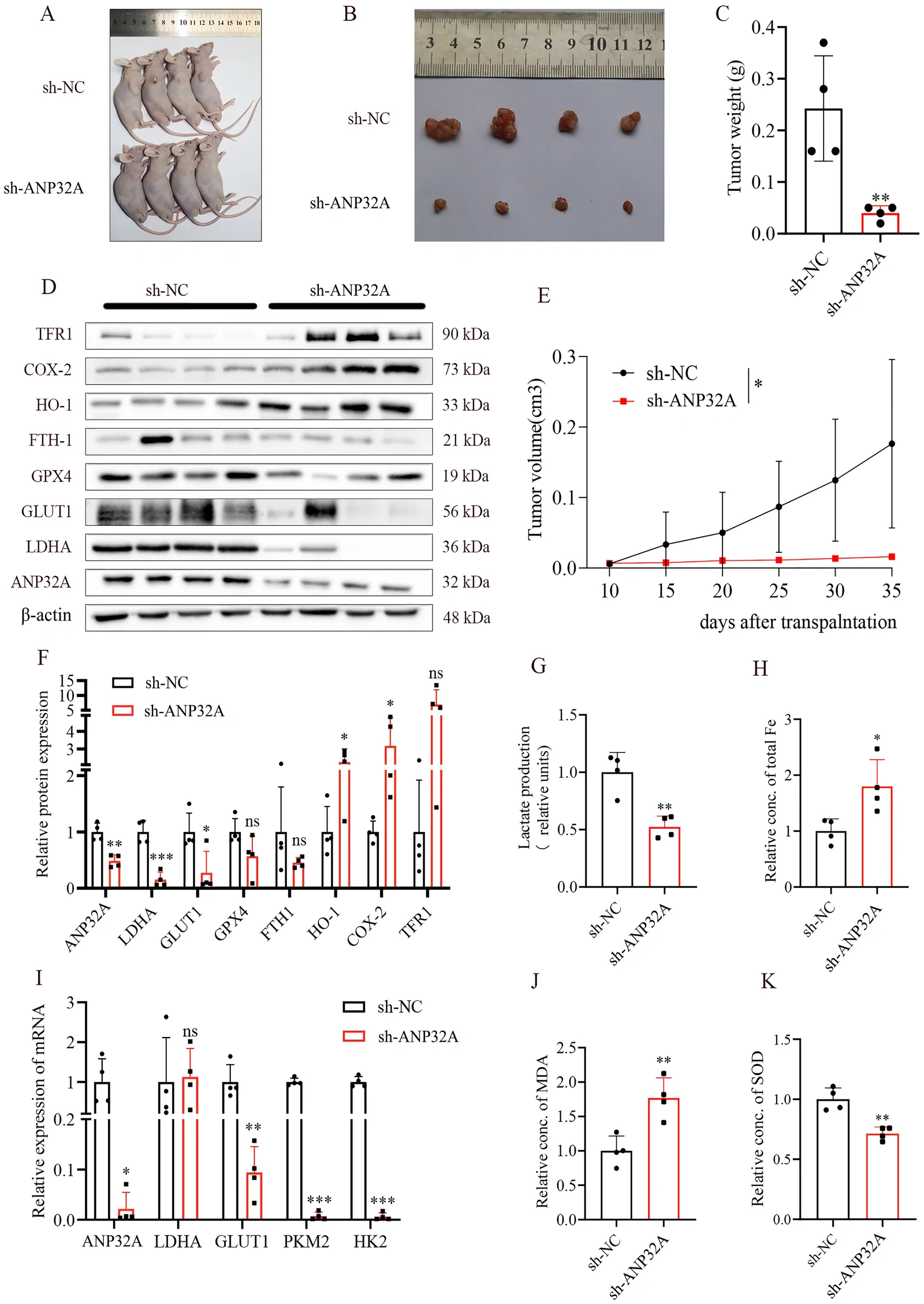
ANP32A depletion *in vivo* suppressed cell proliferation and glycolysis and elicited ferroptosis. **(A, B)** The photographs of nude mice and tumors. **(C, E)** The tumor volume and weight of nude mice. **(D, F)** Western blotting served to detect the expression of TFR1, COX-2, HO-1, FTH1, GPX4, GLUT1, LDHA, and ANP32A in tumor samples. **(G)** Lactate content in tumor tissues. **(H)** Total iron ion content in tumor tissues. **(I)** qPCR was performed to evaluate the mRNA expression levels of ANP32A, LDHA, GLUT1, PKM2, and HK2 in tumor tissues. **(J, K)** The MDA content and SOD activity in tumor tissues. The above values are expressed as mean ± SD, n = 4, *P < 0.05, **P < 0.01, ***P < 0.001, ns, *P* > 0.05, *vs.* sh-NC.

Then we investigated ANP32A’s role in HCC and its effect on glycolysis and ferroptosis via *in vivo* experiments. Two groups of mice were established via random assignment and injected with 6×106 Huh-7 cells, either with stable ANP32A knockdown or an empty vector control. The results showed that ANP32A silencing markedly reduced tumor volume and weight in the nude mice(**Figures 7A–C, E**). We then analyzed glycolysis-related proteins and mRNA, as well as ferroptosis-associated proteins expression in the xenograft tissues. In the ANP32A silencing group, LDHA and GLUT1 protein levels were reduced(**Figures 7D, F**), and the mRNA expression of GLUT1, PKM2, and HK2 was downregulated, while LDHA mRNA levels remained unchanged(**Figure 7I**), aligning with our *in vitro* results. Consequently, lactate levels in the xenografts significantly diminished(**Figure 7G**), indicating a marked reduction in the tumor’s glycolytic capacity following ANP32A silencing.

Simultaneously, in the ANP32A silenced xenografts, HO-1 and COX-2 protein expression levels were markedly elevated, whereas GPX4, FTH1 and TFR1 protein levels showed no statistically significant changes (**Figures 7D, F**). Moreover, total iron ion concentration (**Figure 7H**) and MDA levels were notably increased in the ANP32A silencing group (**Figure 7J**), while SOD (superoxide dismutase) levels significantly decreased (**Figure 7K**). The above results revealed that ANP32A silencing inhibits glycolysis and promote ferroptosis *in vivo*, thereby suppressing tumor’s growth. Taken together, these changes in glycolytic metabolism together with the elevation of iron content and MDA, the reduction of SOD and partial ferroptosis-related protein alterations preliminarily imply that ANP32A depletion suppresses tumor growth *in vivo* possibly by inhibiting glycolysis and driving ferroptosis-associated responses.

## 4 Discussion

HCC is a malignant tumor marked by metabolic reprogramming (35, 36). It is usually associated with chronic liver disease and liver damage, non-alcoholic hepatic steatosis, and prolonged alcohol overuse (6, 37, 38). HCC has complex molecular pathological mechanisms, often accompanied by dysregulation of cell cycle control and abnormal activation of signaling pathways, which affect energy production, cell proliferation, and immune evasion (12, 35, 39). In fact, HCC is known to be highly drug-resistant, and sorafenib, the first-line therapy, extends survival in patients with advanced disease by only about three months (40). Via a comprehensive three-stage screening of multiple databases (TCGA, GEO, and GTEx), including differential expression analysis, univariate COX regression analysis, and WGCNA, combined with proteomic profiling, we identified ANP32A as a novel biomarker linked to poor prognosis in HCC.

Mounting evidence establishes that the abnormal expression of ANP32A serves as a critical gene in tumor development and progression (24, 31, 41). Elevated ANP32A expression indicates poor prognosis in HCC. As a potential biomarker, ANP32A facilitates tumorigenesis by enhancing proliferation, metastasis, and impairing apoptosis. Its involvement in regulating the cell cycle further underscores its significance in the progression of HCC. Elevated levels of ANP32A may contribute to more aggressive tumor behavior and serve as an indicator of unfavorable clinical outcomes (24, 31, 32). As a constituent of the SET complex that is connected with the endoplasmic reticulum, ANP32A functions within the granzyme A-mediated apoptosis pathway (42). Recent research has shown that ANP32A contributes to poor prognosis in various cancers, encompassing colorectal cancer(CRC) and leukemia (27, 28). Our prior studies also revealed that ANP32A exhibits elevated expression in CRC patients and promotes CRC proliferation and EMT capabilities by triggering the PI3K/AKT and ERK signaling pathways (28, 43). This study reveals that ANP32A knockdown suppresses the proliferation of HCC cells and restrains glycolysis in HCC, with ANP32A interacting with LDHA and modulating its ubiquitination to mediate these phenotypic changes.

Glycolysis is a key metabolic pathway distinguishing normal cells from cancer cells, enabling cancer cells to preferentially employ glycolysis for energy, even under aerobic conditions (44, 45). In HCC, a metabolic disease, both glucose and fatty acid metabolism are reprogrammed (12, 46, 47). Interfering with the glycolytic process may be a crucial step toward curing HCC. ANP32A has been recognized as a critical regulator that facilitates the proliferation of liver cancer cells. LDHA mediates aerobic glycolysis through pyruvate-to-lactate conversion in aerobic environments and is a pivotal regulator of the persistent aerobic glycolysis in tumors (48). Distinct from other LDH subunits, LDHA is predominantly expressed in cancer cells, promoting tumorigenesis and enhancing tumor metabolism (49, 50). Research indicates that elevated LDHA expression is strongly related to unfavorable prognosis in HCC (51–53). HIF-1α and c-Myc can individually or cooperatively regulate LDHA transcription (54–56). PPI network predictions combined with GO and KEGG analysis showed that ubiquitination process is one of the primary pathway affected by ANP32A. Ubiquitination is a major post-translational modification in cells, and it can modify substrate proteins through either mono- or poly-ubiquitination (57). Our findings showed that ANP32A directly interacts with LDHA and enhances its protein stability. LDHA is a rate-limiting enzyme of glycolysis that catalyzes the reduction of pyruvate to lactate, thereby serving as a pivotal mechanism for maintaining NAD^+^ recycling in cancer cells (56). Co-IP and immunofluorescence experiments showed that ANP32A binds to LDHA and co-localizes in the cytoplasm, and this interaction is closely associated with the modulation of aerobic glycolysis in HCC cells. Further investigation revealed that silencing ANP32A led to increased ubiquitination and accelerated degradation of LDHA protein. In addition, the combined effect of 2-DG and ANP32A knockdown points to a potential therapeutic avenue for HCC through glycolytic intervention in tumor metabolism. We found that ANP32A binding to LDHA reduces its ubiquitination and proteasomal turnover, suggesting that this association may shield LDHA from recognition and ubiquitin conjugation by E3 ligases. This study suggest that ANP32A forms a functional complex with LDHA, participating in the regulatory network between glycolysis and ferroptosis in HCC. This discovery not only expands the understanding of ANP32A’s biological functions in tumor metabolic regulation but also provides a new therapeutic approach that targets the interface between metabolic intervention and cell death regulation.

In existing studies, the degradation of LDHA has been showed to be significantly linked to ferroptosis and cancer progression (19, 58). Studies have shown that APOL3 overexpression can augment the ubiquitination of LDHA, which in turn promotes ferroptosis induced by the ferroptosis activator RSL3 (19, 59, 60). Furthermore, ANP32A silencing mediated ubiquitination and degradation of LDHA enhanced the tumor’s sensitivity to ferroptosis. However, its involvement in ferroptosis in HCC remains elusive. Relying on iron, ferroptosis represents a distinct mode of cell death, which mainly eliminates cancer cells by inducing lipid peroxidation and disrupting cell membranes. HCC often develops resistance to cisplatin and sorafenib in a short period, which is a major reason for poor prognosis in advanced HCC. Research suggests that enhancing the sensitivity of HCC to ferroptosis could effectively impede the occurrence of resistance in HCC (61, 62). In the current study, we observed a clear accumulation of ROS and notably altered expression of ferroptosis-associated proteins in ANP32A-knockdown HCC cells. Knocking down ANP32A led to an increase in iron ion content. Furthermore, by adding the ferroptosis inducer Erastin, our results suggest that ANP32A showed ferroptosis inhibition in HCC cells. Western blot analysis displayed that ANP32A silencing downregulated the expression of ferroptosis negative regulators (GPX4 and FTH1), while upregulating positive regulators (HO-1 and TFR1). This dual regulatory network suggests that tumor cells dynamically balance proliferation and death through metabolic state sensing.

Based on these findings, we speculate that downregulating ANP32A can enhance ferroptosis in HCC cells. Moreover, we found that either glycolysis inhibitor treatment or ferroptosis inducer all increased the susceptibility of ANP32A silenced HCC cells to ferroptosis induction and glycolysis inhibition, which suggests that ANP32A may affect glycolysis and ferroptosis in HCC.

This research bears certain limitations. Our functional and mechanistic conclusions are drawn primarily from cell line assays and public omics data, without additional validation using independent clinical HCC patient cohorts to confirm the translational relevance of our findings. While we verified that ANP32A curbs ubiquitination-mediated proteasomal breakdown of LDHA to preserve its protein abundance, the specific E3 ubiquitin ligase or deubiquitinating enzyme mediating this process and the exact binding interface between ANP32A and LDHA remain uncharacterized. Furthermore, we have not performed rescue experiments to overexpress LDHA and rescue the ferroptotic phenotypes induced by ANP32A knockdown, which leaves open the possibility of additional, non-LDHA-mediated metabolic effects of ANP32A. These unresolved points will be addressed with complementary experiments and deeper mechanistic dissection in our future investigations.

## 5 Conclusion

In summary, this study suggests that ANP32A interacts with LDHA and is associated with reduced ubiquitination and degradation of LDHA, which may contribute to enhanced glycolysis and ferroptosis resistance in HCC. Overall, this study provides novel insights into the function of ANP32A in HCC and its regulatory mechanisms involving glycolysis and ferroptosis, highlighting the potential therapeutic strategy of inhibiting ANP32A combined with ferroptosis activators or glycolysis inhibitors for treating HCC.

## Data Availability

The public datasets analyzed in this study are available in the TCGA (https://portal.gdc.cancer.gov/), GEO (https://www.ncbi.nlm.nih.gov/geo/), and GTEx (https://gtexportal.org/home/) repositories. The original data generated during this study, including but not limited to cell lines and animal model data, are not publicly available due to laboratory data management policies but are available from the corresponding author upon reasonable request.
