# Supplementary material for: ANP32A interacts with LDHA to modulate glycolysis and ferroptosis in hepatocellular carcinoma
Source: Front Oncol. 2026 Aug 28;16:1908459. doi: 10.3389/fonc.2026.1908459 (PMC13579258; doi:10.3389/fonc.2026.1908459)
Supplement: Supplementary file 1 [file SupplementaryFile1.docx]

**Supplementary Figure 1. ANP32A promotes glycolysis and enhances proliferation in HCC cells. (A, E)** Western blot was used to detect the expression of LDHA and GLUT1 proteins. **(B, F)** RT-qPCR was used to detect the expression of glycolysis-related factor mRNA. **(C-D, G-H)** Glucose uptake and lactate production were measured when ANP32A was knocked down in SK-HEP-1 cells and overexpressed in Huh-7 cells. **(I, L)** Western blot was used to detect the expression of LDHA and GLUT1 proteins after the addition of 2-DG to the transfected cell lines. **(J,M)** Lactate production was measured after adding 2-DG to the transfected cell lines. **(K, N)** MTT was used to assess cell proliferation after adding 2-DG to the transfected cell lines.The internal control was β-actin.The above values are expressed as mean ± SD, *n* = 3, ^*^*p* < 0.05, ^**^*p* < 0.01, ^***^*p* < 0.001, ^ns^ *p* > 0.05.vs. sh-NC or OE-NC; ^#^ *p* < 0.05, ^##^ *p* < 0.01, ^###^ *p* < 0.001, *^ns^* *p* > 0.05, vs. sh-ANP32A+2-DG or OE-ANP32A+2-DG.
